# Report of Committee on the Poor Department of Erie County, N. Y.

**Published:** 1850-11

**Authors:** 


*Report of Committee on the Poor Department of Erie County, N. Y.*—By the published proceedings of the County Supervisors at their late sitting, we observe that the Report of the Superintendents of the Poor shows an improvement in the rate of mortality at the Poor-house during the last year. The number relieved was 1,540, and the number of deaths 155, making a ratio of a fraction over *one* death in *ten* of the whole number received. We take pleasure in chronicling this improvement, which is doubtless owing to some increase in the accommodations, together with more careful and skillful attention to hygienic provisions. There is still, however, abundant need for further amelioration of the condition of the sick at the poor-house. A stirring Report on this subject, and on the evils of the present pauper system, was made by the Committee on the Poor Department (Dr. Baker, of Colden, being a member of the Committee,) and the Board of Supervisors adopted a resolution to purchase a farm and erect buildings more extensive and appropriate than those now occupied.

The mortality among those relieved at their homes in the city, continues to be truly appalling, being, for the past year, 518. We have no data for determining the number of sick persons among this class of paupers. Assuming the ratio of deaths to be the same as at the poor-house, the number of cases calling for medical treatment during the year, is *five thousand one hundred and eighty !*

